# Prevalence of Misconceptions and Myths Regarding Oral Health Among Medical and Dental Students in Peshawar, Pakistan

**DOI:** 10.7759/cureus.52800

**Published:** 2024-01-23

**Authors:** Ahmad Deedar Khan, Mahad Ali, Muhammad Aqeel Ajmal, Farrukh Siyar Wazir, Asim Ahmad, Muhammad Awais, Sofia Kabir

**Affiliations:** 1 Endodontics, Sardar Begum Dental College, Gandhara University, Peshawar, PAK; 2 General Dentistry, Sardar Begum Dental College, Gandhara University, Peshawar, PAK; 3 Dentistry, Sardar Begum Dental College, Gandhara University, Peshawar, PAK; 4 Orthodontics, Sardar Begum Dental College, Gandhara University, Peshawar, PAK; 5 Epidemiology and Public Health, Kabir Institute of Public Health, Sardar Begum Dental College, Gandhara University, Peshawar, PAK

**Keywords:** dentistry, quackery, hygiene, education, awareness, knowledge, oral health

## Abstract

Background

The aim of study is to assess the prevalence of dental myths and misconceptions among the students of medicine and dentistry in Peshawar, Pakistan, and to gauge the quality of dental education, knowledge, and awareness.

Methods

A cross-sectional descriptive study was conducted on the current students of medicine and dentistry at Rehman Medical College, Rehman College of Dentistry, Gandhara University, Khyber Medical College, and Khyber College of Dentistry. They were questioned regarding their beliefs in dental misconceptions and myths prevalent in Peshawar, Pakistan.

Results

The sample comprised 400 undergraduate students, of whom 47.5% (190) were males and 52.5% (210) were females. The most held beliefs were that eating sugar does not affect teeth as long as one brushes twice a day (46%), brushing right after a meal is better for teeth (70%), and the extraction of baby teeth does not matter as they are going to be replaced by the permanent teeth in any case (38%). Most of these misconceptions had a significant association with the field of study.

Conclusion

The issue of myths and misconceptions regarding dental health and care is widespread, even in the academic community. This should be of significant concern to the relevant authorities, and adequate measures must be taken to dispel such false information.

## Introduction

Oral health is such an essential indicator of general well-being, yet it is overlooked just as much. Substandard oral health might cause dejection and a deteriorated social presence, and even go as far as chronic stress and depression. Thus, the statement that "Oral health status reflects the general health and quality of life" seems appropriate [1]. Oral diseases are significantly prevalent all over the globe, with a direct correspondence to low awareness and knowledge about oral health [2].

There has been a significant advancement in the quality of dental care over the past century; however, a considerable proportion of the population has held themselves back from getting the appropriate care due to the prevalence of age-old myths passed down from generation to generation.

“Myth” originates from the Greek word "mythos," which has varied meanings, including “word,” “story,” or “fiction” [3]. Both the secular and religious factions usually affirm them, having close ties with religion and spirituality, sometimes considered part of religion [4]. The problem of myths in the field of dentistry is a grave one. Many myths circulate around the globe, still being regarded as sacred truths, plenty of which relate to extraction. Over half the people surveyed in Chennai, India, believed that these extraction-related myths were true [5].

Similarly, a study on the same issue in Islamabad, Pakistan, concluded that dental myths are a significant concern that needs to be tackled head-on. The prevalence of these myths and misconceptions was associated with a lack of education, and some variance in gender was found as well, with females being more prone to believing in them [6]. These sentiments are corroborated by another research conducted in Bhopal, India, which confirmed a vast difference between the actual science behind oral health and what the masses believe [7].

These myths lead to people taking inappropriate measures regarding concerns over their oral health, leading to a deteriorated health status. In poorly developed countries like Pakistan, the ever-growing problem of quackery only helps to exacerbate the issue.

Pakistan can be classified as a poorly developed country, ranked 154 out of 189 countries [8], with a gross national income per capita of $1,280 as of 2020 [9] and around 39.2% of people living below the poverty line in 2021 [10]. Most concerning is that Pakistan ranks 150 among the 163 countries in the education sector [11].

Considering these factors, it would be safe to assume that the quality of education in Pakistan is below par. The prevalence of these myths has been well documented among the poor and uneducated [12]; however, a study has yet to be carried out concerning its prevalence among the educated masses. Pakistan is a religious nation with solid beliefs in supernatural phenomena, and therefore these myths hold strong here. Trust in faith healers and black magicians is widespread [13]. Combining this with the poor quality/non-existent dental education in schools and colleges [14], one would not be wrong to think that an average educated Pakistani may still believe in these myths.

The current study aims to assess the prevalence of dental myths and misconceptions among the students of medicine and dentistry in Peshawar, Pakistan.

## Materials and methods

Study design

A cross-sectional descriptive study was conducted on the current students of medicine and dentistry in Rehman Medical College, Rehman College of Dentistry, Gandhara University, Khyber Medical College, and Khyber College of Dentistry from March 1, 2022, to April 15, 2022. Convenience sampling was used while choosing the participants.

Sample size

The sample size was calculated using the online calculator.net sample size calculator [15]. The sample size was based on a population of 10,000. Using a 95% confidence level, a 5% margin of error, and a population proportion of 50%, the required sample size came out to be 370. A total of 400 participants were included in the study to account for any missing data.

Eligibility criteria

Inclusion criteria encompassed all medicine and dentistry students enrolled in the undergraduate program at the time of study, ensuring a thorough representation of the target population. The aim was to get an equal representation of each institution in the study. Thus, 64 participants represented Sardar Begum Dental College, Khyber Medical College, Rehman Medical College, and Khyber College of Dentistry, 68 participants represented Rehman College of Dentistry, and 76 participants represented Kabir Medical College.

Research instrument

**Figure 1:**
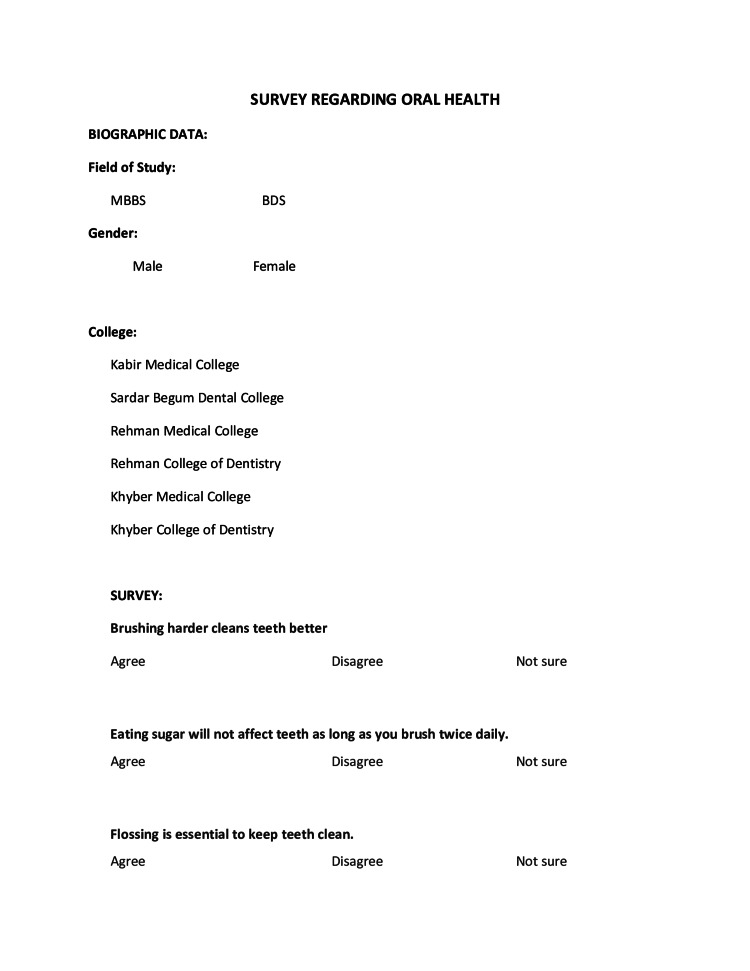
Questionnaire presented to the participants MBBS, Bachelor of Medicine and Bachelor of Surgery; BDS, Bachelor of Dental Surgery

**Figure 2:**
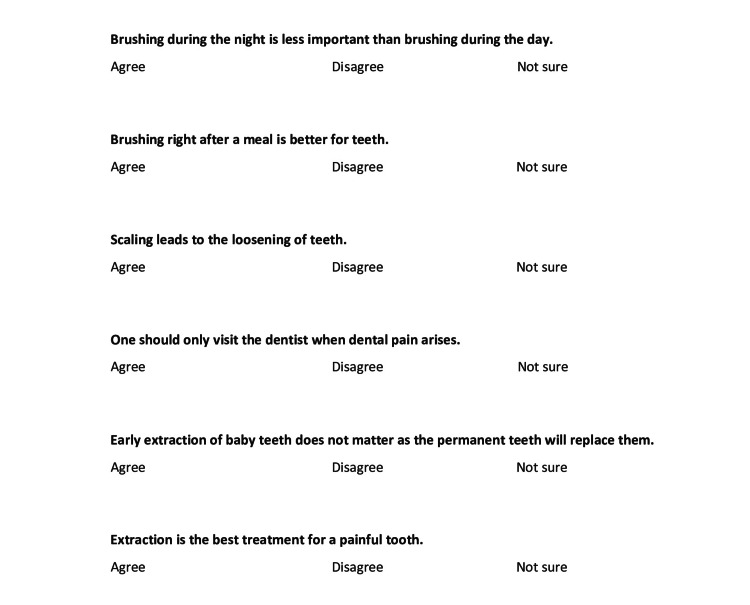
Questionnaire presented to the participants

A questionnaire was designed consisting of two sections. The first section was to collect the participants' sociodemographic data, while the second section consisted of nine close-ended statements regarding common dental misconceptions. The statements were prepared with the help of available literature [12,14,16] and prevalent myths based on data collected by surveys carried out in the region previously. A pilot survey was conducted to test for content validation before administering it to the participants. The questionnaire was put forth to experts in the field as well. The statements put forth to the participants are shown in Figure 1 and Figure 2.

Information was collected by visiting the universities mentioned above and distributing the questionnaire among students as well as sharing an online questionnaire that the participants voluntarily filled out. All statistical analyses were carried out using SPSS Version 26 (IBM Corp., Armonk, NY). Descriptive statistical analysis was carried out, and a chi-square test was used to determine any association of the gender and field of study of the participants with their responses. A p-value of <0.05 was considered statistically significant.

Ethical considerations

Ethical approval for the survey was taken from the Ethics committee of Gandhara University. The participants were fully informed about the purpose of the survey, and informed consent was obtained. Furthermore, the participants were assured that their data would not be shared with any third party.

## Results

Out of the total study population of 400 undergraduate students, 47.5% (190) were males and 52.5% (210) were females. Kaviya et al. reported that no significant association was found between gender and belief of myths related to dental extractions [16], which is in agreement with the results of our study.

Based on the field of study, our population consisted of 49% (196) students of medicine and 51% (204) students of dentistry. Of the total respondents, 93% (372) belonged to the middle class, 3.5% (14) belonged to the upper class, and 3.5% (14) belonged to the lower class. This classification was made based on the perceptions of participants regarding their socioeconomic status. Sardar Begum Dental College, Khyber College of Dentistry, Rehman Medical College, and Khyber College of Dentistry had 64 participants each. Rehman College of Dentistry had 68 participants, while Kabir Medical College was represented by 76 participants.

**Figure 3:**
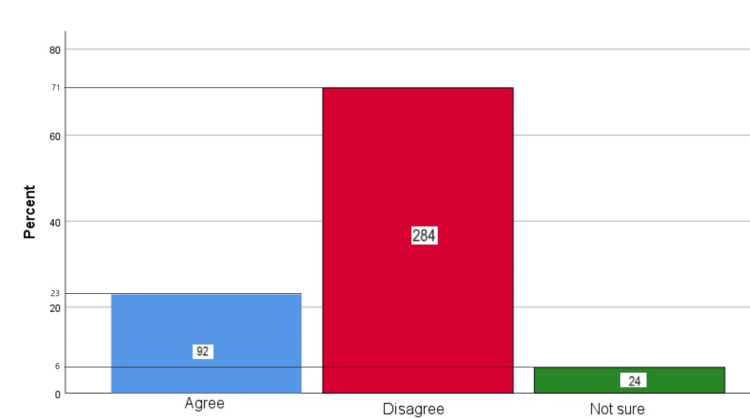
Brushing harder cleans teeth better The bar chart represents the participants' responses regarding their belief that brushing harder cleans teeth better. The total responses are mentioned within each bar.

Figure 3 represents the participants' responses regarding their belief that brushing harder cleans teeth better. Overall, 23% (92) participants believed that brushing harder cleans the teeth better, 71% (284) participants believed that brushing harder does not clean teeth better, and 6% (24) participants were unsure.

**Figure 4:**
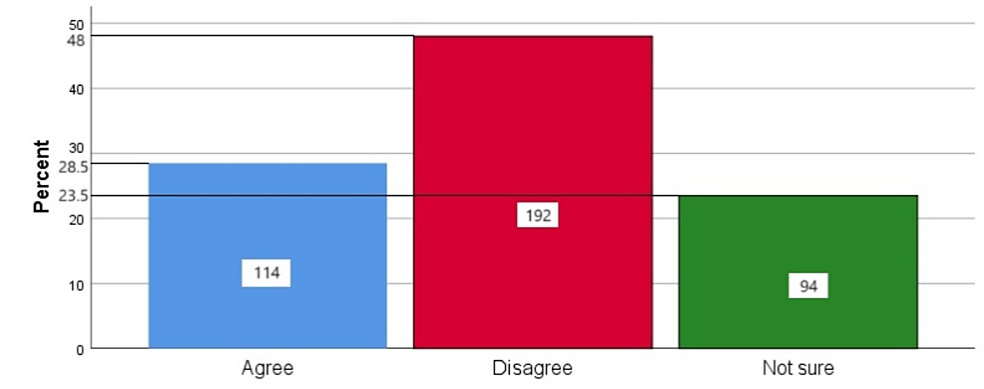
Scaling leads to loosening of teeth The bar chart represents the participants' responses regarding their belief that scaling leads to the loosening of teeth. The total responses are mentioned within each bar.

Figure 4 represents the participants' responses regarding their belief that scaling leads to loosening of teeth. Overall, 28.5% (114) participants believed that scaling leads to the loosening of teeth, 48% (192) participants disagreed with this belief, and 23.5% (94) participants were unsure.

**Figure 5:**
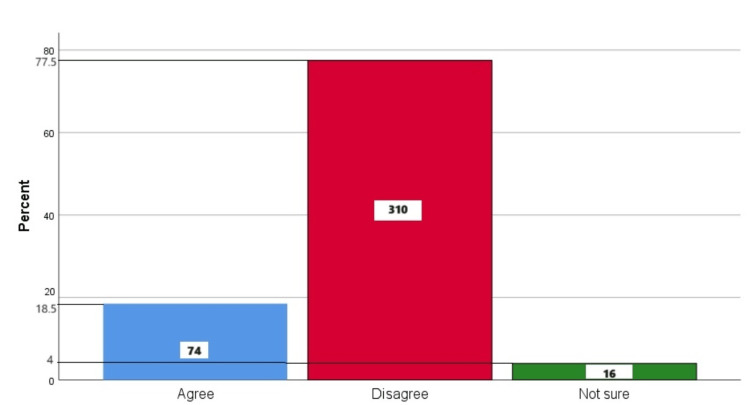
Extraction is the best treatment for a painful tooth The bar chart represents the participants' responses regarding their belief that the best treatment for a painful tooth is extraction. The total responses are mentioned within each bar.

Figure 5 represents the participants' responses regarding their belief that extraction is the best treatment for a painful tooth. Overall, 18.5% (74) participants believed that the best treatment for a painful tooth is extraction, 77.5% (310) participants disagreed with this belief, and 4% (16) participants were unsure.

**Figure 6:**
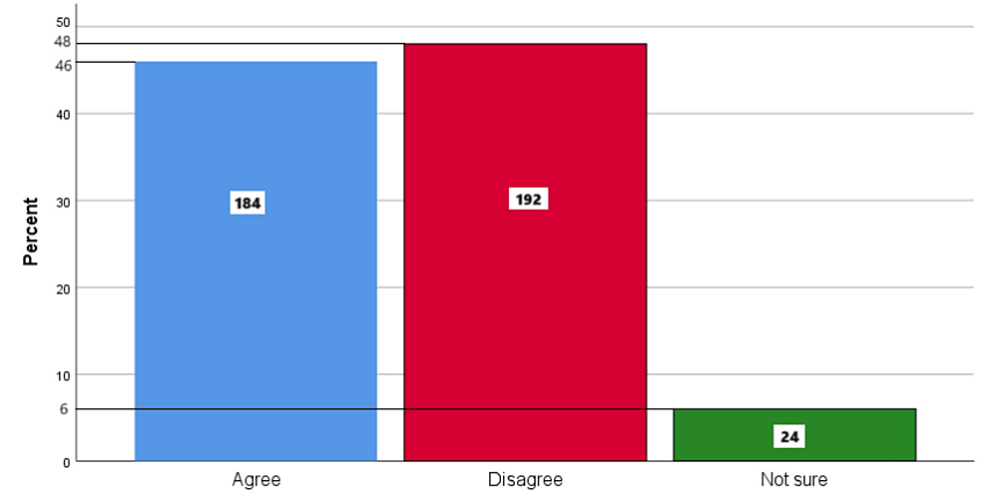
Eating sugar will not affect your teeth as long as you brush twice a day The bar chart represents the participants' responses regarding their belief that eating sugar will not affect their teeth as long as they brush twice a day. The total responses are mentioned within each bar.

Figure 6 represents the participants' responses regarding their belief that eating sugar will not affect their teeth as long as they brush twice daily. Overall, 46% (184) participants believed that sugar will not affect your teeth as long as you brush twice a day, 48% (192) participants disagreed with this belief, and 6% (24) participants were unsure.

**Figure 7:**
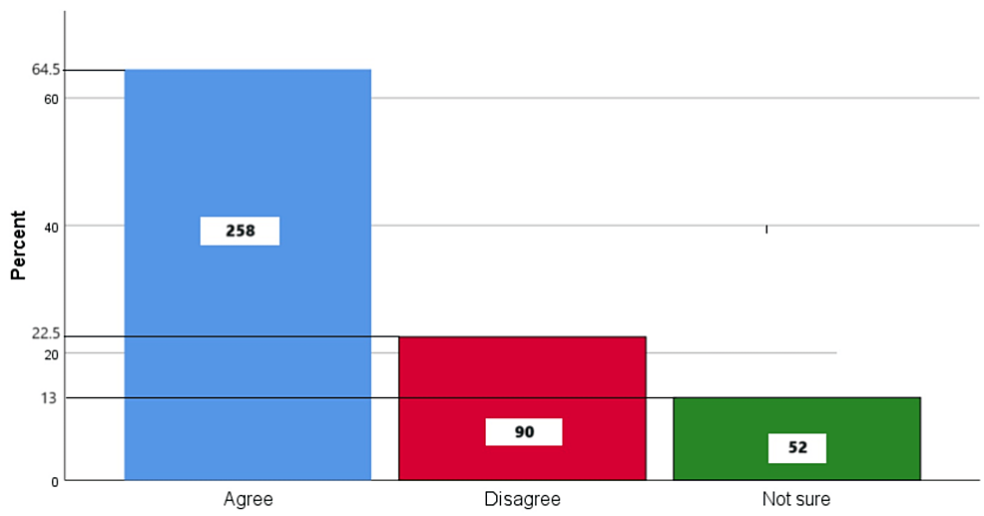
Flossing is important to keep your teeth clean The bar chart represents the participants' responses regarding their belief that flossing is important to keep their teeth clean. The total responses are mentioned within each bar.

Figure 7 represents the participants' responses regarding their belief that flossing is important to keep their teeth clean. Overall, 64.5% (258) participants believed that flossing is important to keep your teeth clean, 22.5% (90) participants disagreed with this belief, and 13% (52) participants were unsure.

**Figure 8:**
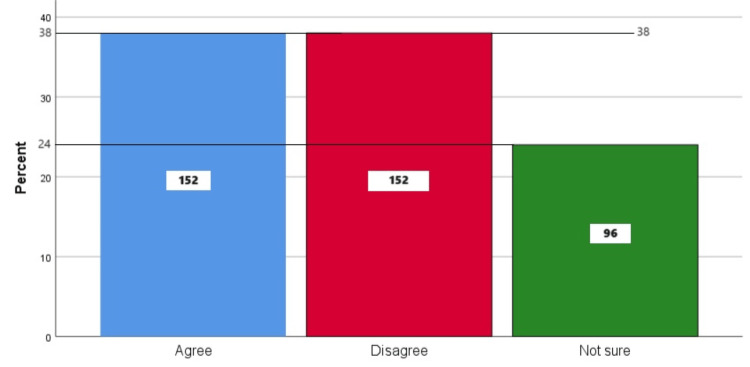
Early extraction of baby teeth does not matter as permanent teeth will replace them anyway The bar chart represents the participants' responses regarding their belief that early extraction of baby teeth does not matter as they will be replaced by permanent teeth anyway. The total responses are mentioned within each bar.

Figure 8 represents the participants' responses regarding their belief that early extraction of baby teeth does not matter as the permanent teeth will replace them anyway. Overall, 38%(152) participants believed that early extraction of baby teeth does not matter as the permanent teeth will replace them anyway, 38% (152) participants disagreed with this belief, and 24% (96) participants were unsure.

**Figure 9:**
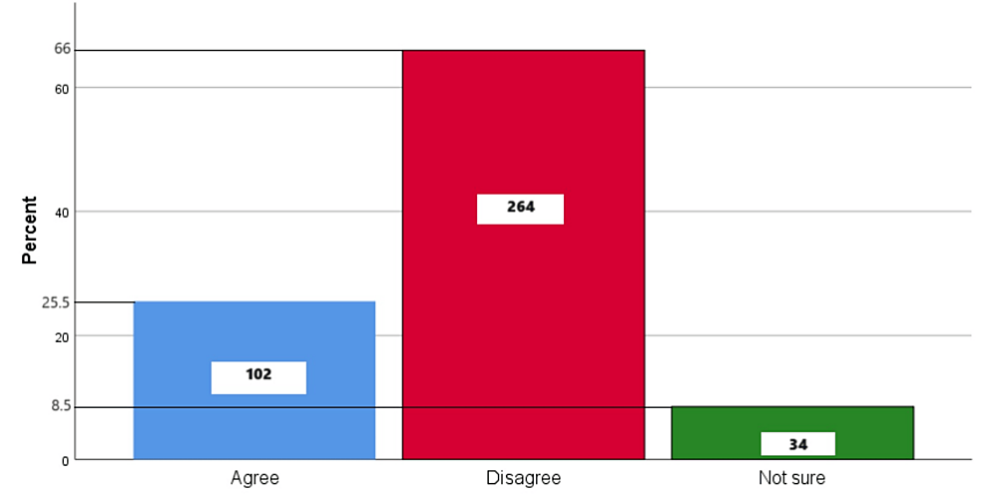
You should only visit the dentist when dental pain arises The bar chart represents the participants' responses regarding their belief that they should only visit the dentist when dental pain arises. The total responses are mentioned within each bar.

Figure 9 represents the participants' responses regarding their belief that they should only visit the dentist when dental pain arises. Overall, 25.5% (102) participants believed that you should only visit the dentist when dental pain arises, 66% (264) participants disagreed with this belief, and 8.5% (34) participants were unsure.

**Figure 10:**
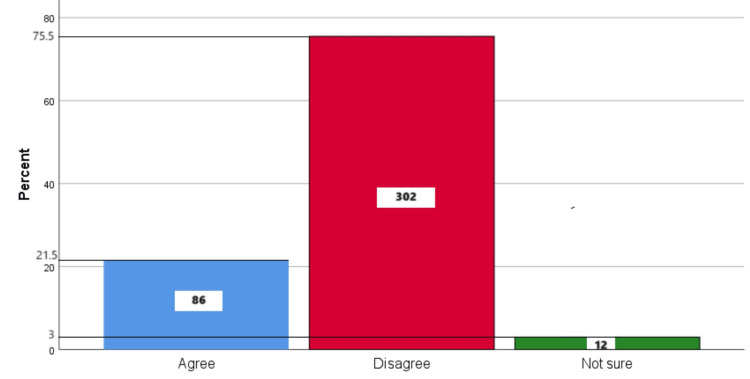
Brushing during the night is not as important as brushing during the day The bar chart represents the participants' responses regarding their belief that brushing during the night is not as important as brushing during the day. The total responses are mentioned within each bar.

Figure 10 represents the participants' responses regarding their belief that brushing during the night is not as important as brushing during the day. Overall, 21.5% (86) participants believed that brushing during the night is not as important as brushing during the day, 75.5% (302) participants disagreed with this belief, and 3% (12) participants were unsure.

**Figure 11:**
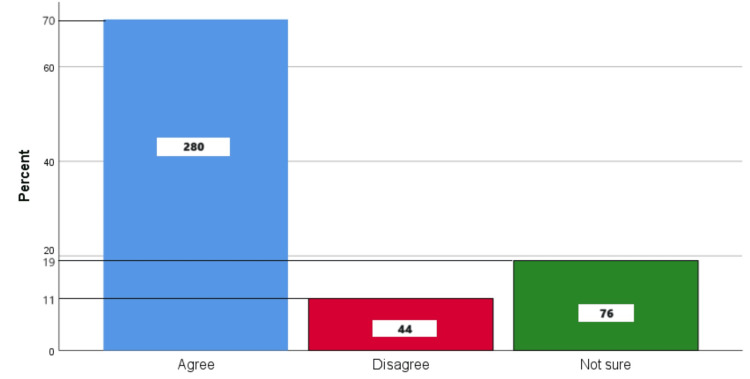
Brushing right after a meal is better for teeth The bar chart represents the participants' responses regarding their belief that brushing right after a meal is better for teeth. The total responses are mentioned within each bar.

Figure 11 represents the participants' responses regarding their belief that brushing right after a meal is better for teeth. Overall, 70% (280) participants believed that brushing right after a meal is better for teeth, 11% (44) participants disagreed with this belief, and 19% (76) participants were unsure.

**Figure 12:**
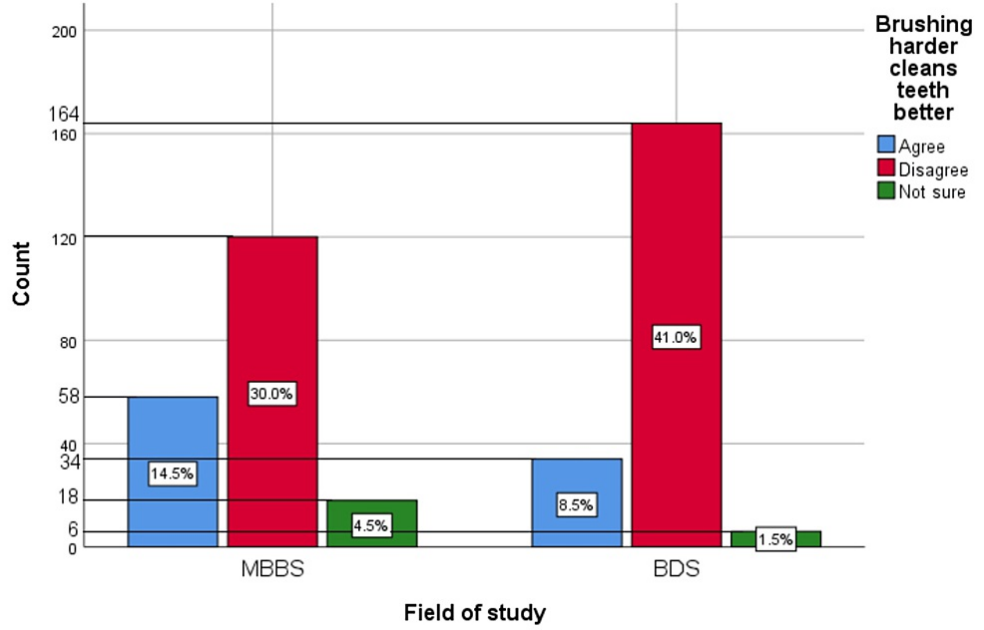
Association of the participants' responses with their field of study The X-axis represents the field of study, while the Y-axis represents the participant count. The percentage of each response is mentioned within their respective bars. p-value=0.009 MBBS, Bachelor of Medicine and Bachelor of Surgery; BDS, Bachelor of Dental Surgery

Figure 12 represents the association of participants' responses regarding the statement “Brushing harder cleans teeth better” with their field of study. The chi-square test showed a significant association of the response with the participant's field of study (p=0.009).

**Figure 13:**
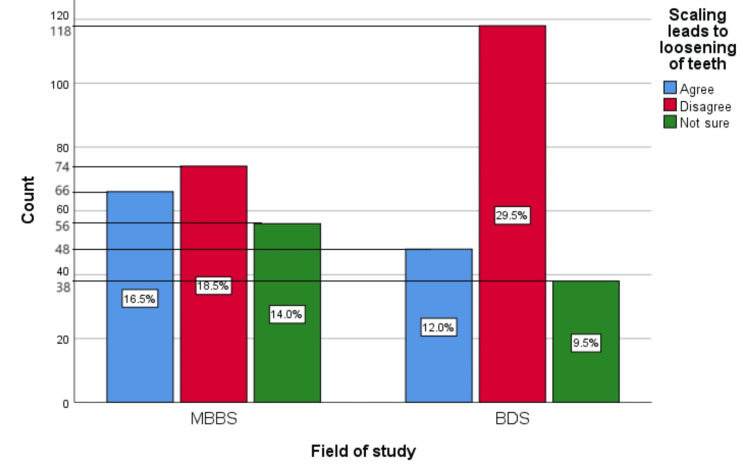
Association of participants' responses with their field of study The X-axis represents the field of study, while the Y-axis represents the participant count. The percentage of each response is mentioned in their respective bars. p-value=0.017 MBBS, Bachelor of Medicine and Bachelor of Surgery; BDS, Bachelor of Dental Surgery

Figure 13 represents the association of participants' responses regarding the statement “Scaling leads to loosening of teeth” with their field of study. The chi-square test showed a significant association of the response with the participant's field of study (p=0.017). A considerable portion of the population found this myth to be true or were unsure about it.

**Figure 14:**
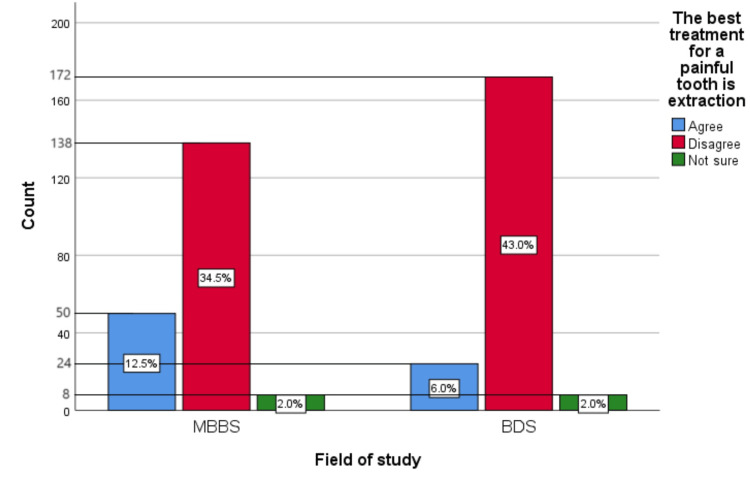
Association of participants' responses with their field of study The X-axis represents the field of study, while the Y-axis represents the participant count. The percentage of each response is mentioned in their respective bars. p=0.042 MBBS, Bachelor of Medicine and Bachelor of Surgery; BDS, Bachelor of Dental Surgery

Figure 14 represents the association of participants' responses regarding the statement “The best treatment for a painful tooth is extraction” with their field of study. The chi-square test showed a significant association of the response with the participant's field of study (p=0.042). Most of the participants found this myth to be untrue.

**Figure 15:**
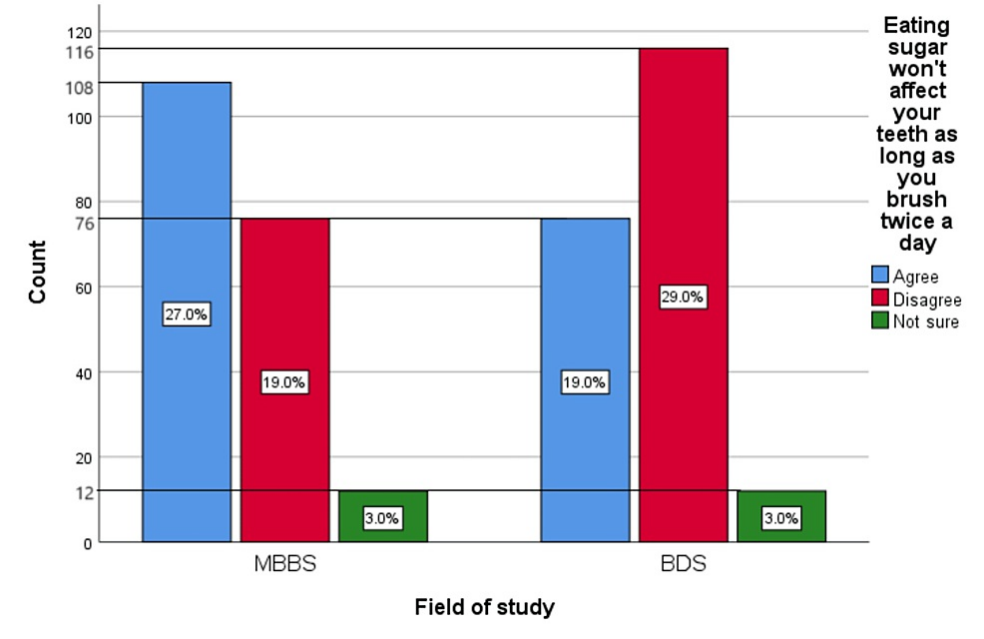
Association of participants' responses with their field of study The X-axis represents the field of study, while the Y-axis represents the participant count. The percentage of each response is mentioned in their respective bars. p-value=0.032 MBBS, Bachelor of Medicine and Bachelor of Surgery; BDS, Bachelor of Dental Surgery

Figure 15 represents the association of participants' responses regarding the statement “Eating sugar will not affect your teeth as long as you brush twice a day” with their field of study. The chi-square test showed a significant association of the response with the participant’s field of study (p=0.032).

**Figure 16:**
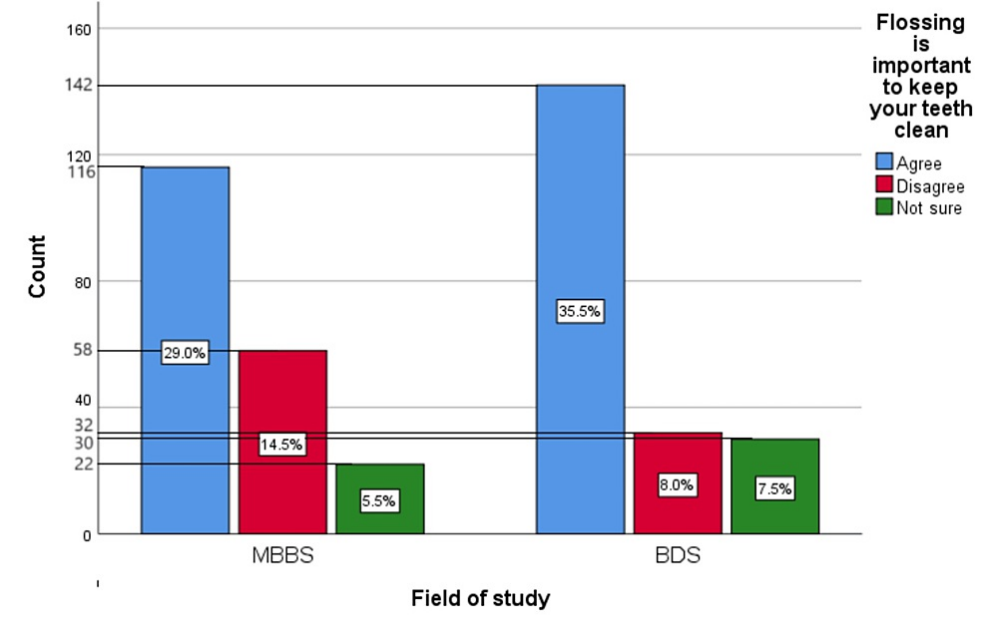
Association of participants' responses with their field of study The X-axis represents the field of study, while the Y-axis represents the participant count. The percentage of each response is mentioned in their respective bars. p-value=0.061 MBBS, Bachelor of Medicine and Bachelor of Surgery; BDS, Bachelor of Dental Surgery

Figure 16 represents the association of participants' responses regarding the statement “Flossing is important to keep your teeth clean” with their field of study. The chi-square test showed no significant association of the response with the participant's field of study (p=0.061).

**Figure 17:**
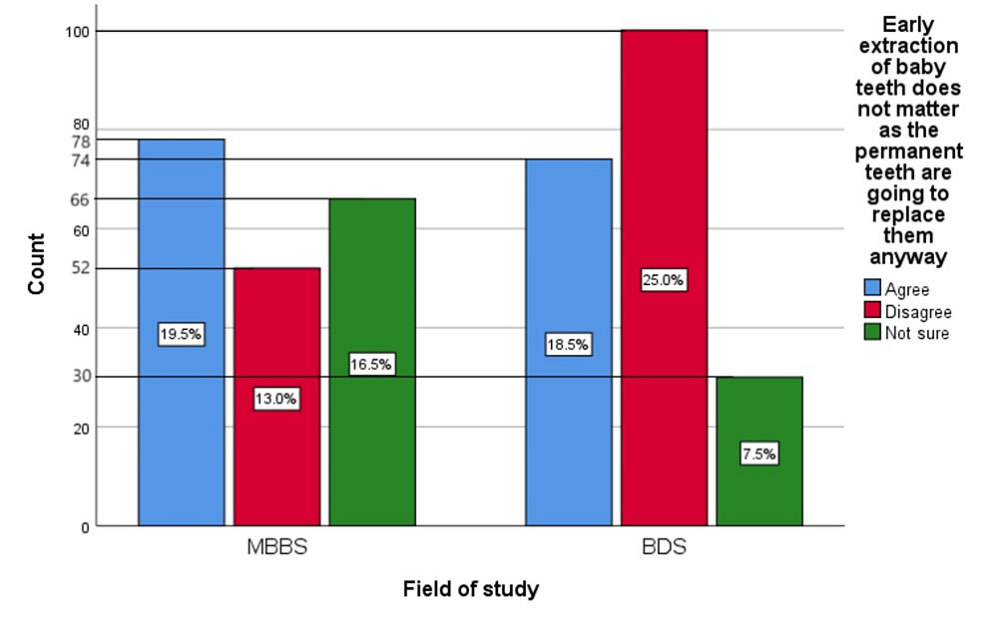
Association of participants' responses with their field of study The X-axis represents the field of study, while the Y-axis represents the participant count. The percentage of each response is mentioned in their respective bars. p-value=0.001 MBBS, Bachelor of Medicine and Bachelor of Surgery; BDS, Bachelor of Dental Surgery

Figure 17 represents the association of participants' responses regarding the statement, "Early extraction of baby teeth does not matter as the permanent teeth are going to replace them anyway” with their field of study. The chi-square test showed a significant association of the response with the participant's field of study (p=0.001).

**Figure 18:**
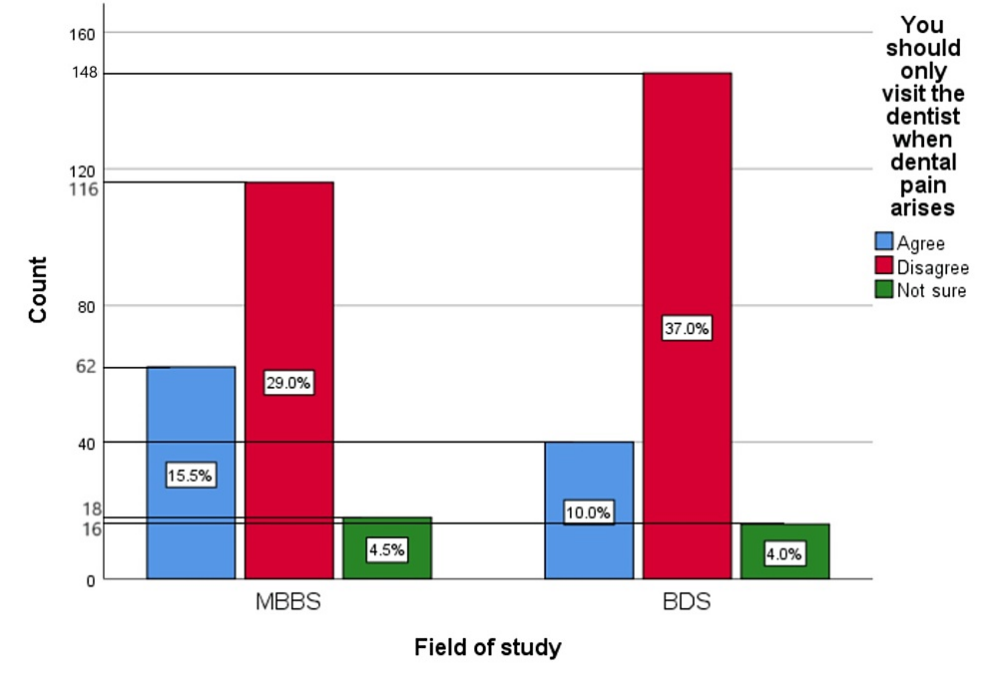
Association of participants' responses with their field of study The X-axis represents the field of study, while the Y-axis represents the participant count. The percentage of each response is mentioned in their respective bars. p-value=0.117 MBBS, Bachelor of Medicine and Bachelor of Surgery; BDS, Bachelor of Dental Surgery

Figure 18 represents the association of participants' responses regarding the statement “You should only visit the dentist when dental pain arises” with their field of study. The chi-square test showed no significant association of the response with the participant's field of study (p=0.117).

**Figure 19:**
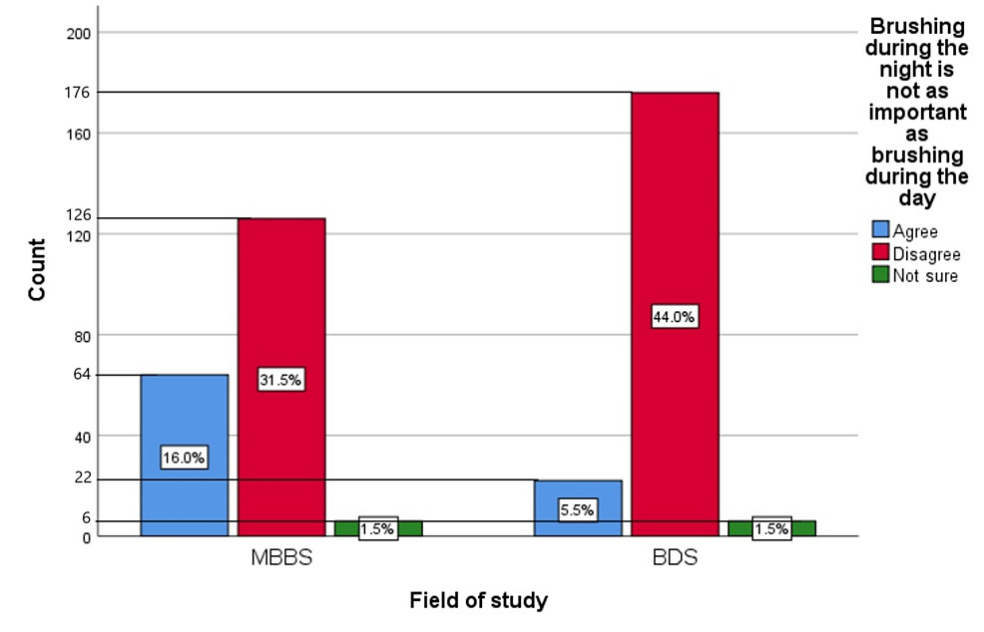
Association of participants' responses with their field of study The X-axis represents the field of study, while the Y-axis represents the participant count. The percentage of each response is mentioned in their respective bars. p-value=0.001 MBBS, Bachelor of Medicine and Bachelor of Surgery; BDS, Bachelor of Dental Surgery

Figure 19 represents the association of participants' responses regarding the statement “Brushing during the night is not as important as brushing during the day” with their field of study. The chi-square test showed a significant association of the response with the participant's field of study (p=0.001).

**Figure 20:**
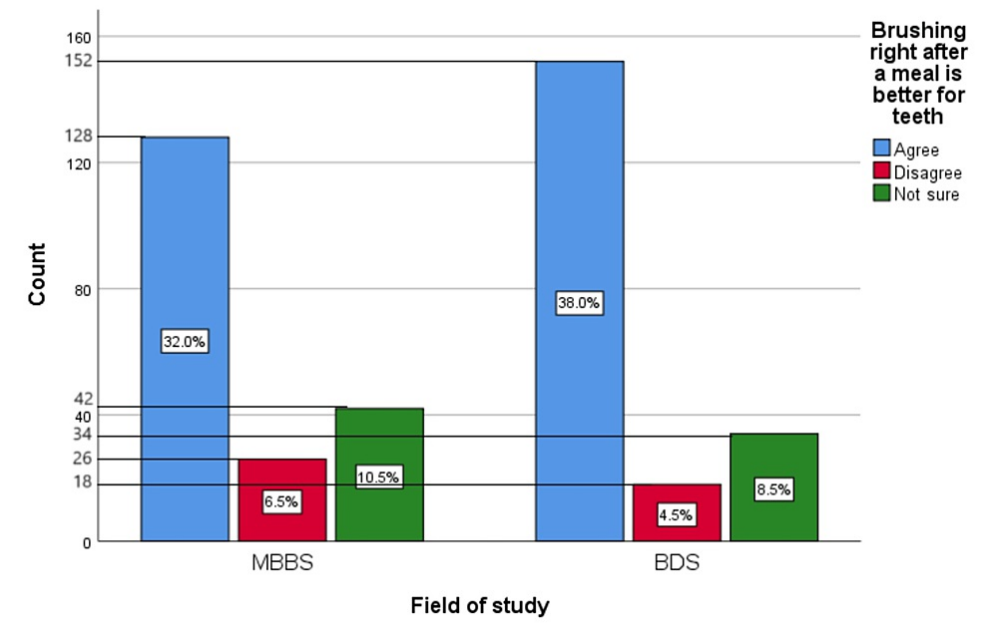
Association of participants' responses with their field of study The X-axis represents the field of study, while the Y-axis represents the participant count. The percentage of each response is mentioned in their respective bars. p-value=0.035 MBBS, Bachelor of Medicine and Bachelor of Surgery; BDS, Bachelor of Dental Surgery

Figure 20 represents the association of participants' responses regarding the statement “Brushing right after a meal is better for teeth” with their field of study. The chi-square test showed no significant association of the response with the participant's field of study (p=0.035). Most of the participants on both the dental and medical sides showed a strong belief in this myth.

The relationship between the gender of participants and the prevalence of misconceptions and myths regarding oral health and treatment was also analyzed; however, no significant association was found. The only aspect of oral health where a significant association was found was the importance of flossing to keep the dentition clean (p=0.000).

## Discussion

Being a developing country, Pakistan suffers from the plight of a heightened disease burden and a minimal budget to be equipped with the treatment needs of the population [17], which predisposes to an increase in the number of patients looking for cheaper alternatives to the costly dental care they need. This, in turn, leads to a significant portion of the population opting for quacks instead of qualified dental professionals. These quacks further reinforce these misconceptions, exacerbating the problem. Faiq et al. reported that the problem of quackery was associated with the issues of affordability, lack of knowledge, awareness, time, and availability of government dental hospitals [18].

Pakistan suffers from a poor educational status on the global level and a high level of unemployment, which correspond statistically to false beliefs regarding oral health. This statement has been corroborated by several studies conducted in the past, both in Pakistan and at the global level [5,6].

Despite the gradual, continuous advancement in the education sector in Pakistan [19], it is evident from the study that the Department of Dental Education needs to be more robust in eradicating the false perceptions of the population regarding oral health. Most of the population has yet to mature psychologically or spiritually enough to seek knowledge about unknown phenomena from reliable sources in books or mass media. People would rather gossip about others and events than seek knowledge, believing in word of mouth and others' logic rather than checking out the facts [20]. These findings can be applied to the population of Pakistan as well, given the results of our study, which shows that even the most educated of Pakistanis, at the peak of their educational status in the country, still believe most of the myths presented in the study to be true.

Most of the studies conducted in the past have either targeted people of low socioeconomic status as their study population or attributed the belief in these myths to low socioeconomic status and poor education [12]. Thus, this study focused on the highly educated segment of Pakistani society to analyze whether Pakistan's education system has done enough to dispel these myths. This study's results show that that is not the case. The study population is people studying in some of the country's most prestigious educational institutes. Nevertheless, the belief in these myths is quite prevalent among those not associated with the dental community. This false perception of the population is found in regard to even the basic facts, such as believing that visiting the dentist is only necessary when dental pain arises and having no knowledge of the negative consequences of extracting the deciduous dentition rather than letting them shed naturally.

These findings are a grave concern for Pakistan's educational and health institutes and the dental community. The educational sector needs to focus on reshaping the minds of the newer generation to think more critically than the generations before them and to actively seek out knowledge from credible sources instead of believing in hearsay. Hearsay leads to the spread of misinformation, a massive problem in Pakistan. It has led to people hoarding medicine during the COVID outbreak [21] and an increase in polio cases [22]. This suggests that the problem of misconceptions is not specific to oral health. Thus, a broader approach must be taken to reform the population's minds.

Furthermore, strict action needs to be taken against the problem of quackery that is rampant in Pakistan. Mustufa et al. reported that 70% of the health practitioners inspected were unlicensed [23]. The issue of quackery is quite a significant one in dentistry as well [24]. Quackery has many negative consequences on the population's oral health, including the spread of misinformation through an authoritative figure. Quacks are barely educated regarding oral health, and when the population turns to them, it only leads to a further deterioration of the situation.

For the dental community, misinformation and misconceptions regarding oral health must be tackled head-on at the early stages of life before they become engraved in their minds. Regular dental camps and seminars need to be held at schools and colleges to educate the population regarding oral health and dispel any myth that leads to a deterioration of their health. The importance of good oral hygiene and regular dentist visits must be repeatedly etched into their minds. The disastrous consequences of quackery need to be highlighted in every forum, and every step needs to be taken to convince the masses to visit only a qualified professional for their dental needs.

Even though the participants associated with the dental field scored significantly better than those associated with medicine, responses to specific questions showed that the education department of dentistry needs to improve in dispelling these myths. Most of the participants in the field of dentistry agreed with the myth that brushing right after a meal is better for teeth. Similarly, many dental students fail to grasp the significance of timely shedding of healthy milk teeth in the normal oral development of a child. Quite a few dental students also believed that sugar would not harm teeth as long as one brushes twice daily. This shows that either our education department needs to be improved severely or these beliefs are instilled too deeply into the younger generation.

Limitations

The scope of the study was limited to certain institutions easily accessible to the authors. The year of study of the participants was not recorded, and the study was limited to a single city.

## Conclusions

Myths and misconceptions regarding oral health and treatment have a disastrous effect on the general population's knowledge, attitude, and health-seeking behavior. These myths prove to be a significant barrier to seeking dental care. The purpose of this study was to assess the prevalence of myths and misconceptions regarding oral health among medical and dental students in Peshawar. The results of the study show that, overall, students lacked the knowledge required to keep their oral health in check. This shows the insufficiency of institutions in providing proper dental education to students. In such a scenario, even if the government provides affordable and accessible healthcare, the belief in these myths acts as a hindrance. If the population is educated and made aware of the proper knowledge regarding prevention and cure, these myths will be eradicated, and a healthier community can be our future. Basic oral health and hygiene awareness must be part of the educational curriculum starting at the elementary level to dispel these ideas before they take root in the younger generations' minds. On a broader level, dental health education campaigns need to be a part of the health and education departments to tackle these false perceptions head-on. Access to correct knowledge should be provided far and wide to eradicate these myths and get people to start caring about their health and hygiene.
